# Cell-Membrane-Coated Metal–Organic Framework Nanocarrier Combining Chemodynamic Therapy for the Inhibition of Hepatocellular Carcinoma Proliferation

**DOI:** 10.3390/pharmaceutics16050619

**Published:** 2024-05-05

**Authors:** Huaying Xie, Xuhua Xiao, Xiaoyuan Yi, Kunzhao Huang, Liyan Wang

**Affiliations:** 1 The First School of Clinical Medicine, Guilin Medical University, Guilin 541006, China; xiehuaying@stu.glmc.edu.cn (H.X.); yixiaoyuan@glmc.edu.cn (X.Y.); huangkunzhao@stu.glmc.edu.cn (K.H.); 2 Department of Gastroenterology, Affiliated Hospital of Guilin Medical University, Guilin 541001, China; xiaoxuhua@glmc.edu.cn

**Keywords:** chemodynamic therapy, metal–organic framework, HIF-1α inhibition, cell membrane

## Abstract

Chemodynamic therapy (CDT) employs hydrogen peroxide (H_2_O_2_) within the tumor microenvironment (TME) to initiate the Fenton reaction and catalyze the generation of hydroxyl radicals (·OH) for targeted therapy. Metal ion-based nanomaterials have garnered significant attention as catalysts due to their potent anti-tumor effects. Hypoxia in the TME is often associated with cancer cell development and metastasis, with HIF-1α being a pivotal factor in hypoxia adaptation. In this study, an organic framework called MIL-101 (Fe) was designed and synthesized to facilitate H_2_O_2_-induced ·OH production while also serving as a carrier for the HIF-1α inhibitor Acriflavine (ACF). A biomimetic nanomedical drug delivery system named MIL-101/ACF@CCM was constructed by encapsulating liver cancer cell membranes onto the framework. This delivery system utilized the homologous targeting of tumor cell membranes to transport ACF, inhibiting HIF-1α expression, alleviating tumor hypoxia, and catalyzing ·OH production for effective tumor eradication. Both in vivo and in vitro experiments confirmed that combining ACF with chemotherapy achieved remarkable tumor inhibition by enhancing ROS production and suppressing HIF-1α expression.

## 1. Introduction

Hepatocellular carcinoma (HCC) is a prevalent malignancy worldwide, characterized by a high incidence, mortality, and recurrence rate [1]. Despite a wide range of treatment options being available, such as radical resection, liver transplantation, radiotherapy, and chemotherapy, their therapeutic effectiveness remains unsatisfactory [2,3]. The primary factor influencing the prognosis of liver cancer is the high rate of postoperative recurrence caused by intrahepatic recurrence and metastasis [4,5]. Furthermore, the acidic microenvironment and microvascular invasion in tumor tissue pose challenges in the targeted therapy of HCC [6,7]. The pathogenesis of HCC is multifaceted, and the prognosis is unfavorable. Current clinical monotherapy or therapeutic drugs alone are insufficient to achieve a therapeutic effect [8]. Therefore, there is an urgent need to explore novel therapeutic pathways for HCC. The tumor microenvironment (TME) contains hydrogen peroxide (H_2_O_2_) that can catalyze the production of hydroxyl radicals (·OH) through the Fenton reaction [9]. However, recent studies have revealed a close association between TME hypoxia and the development and metastasis of cancer cells, with hypoxia-inducible factor-1 alpha (HIF-1α) playing a key role in hypoxic adaptation [10]. The expression level of HIF-1α increases with the severity of hypoxia in tumor tissue. HIF-1α is known to regulate apoptosis, cell proliferation, and the glycolytic pathway, enabling tumors to survive under hypoxic conditions and promoting their growth, proliferation, and metastasis [11,12,13]. The inhibition of HIF-1α expression may serve as an effective strategy to suppress tumor growth and metastasis, prompting the investigation of HIF-1α inhibitors. Acriflavine (ACF) has been identified as the most potent inhibitor of HIF-1 among the 336 drugs approved by the US Food and Drug Administration (FDA). Its utilization can effectively disrupt the activity of HIF-1α and enhance the body’s tumor-suppressing capability [14,15]. Nevertheless, the intravenous administration of ACF has shown limited efficacy, mainly due to poor tumor accumulation. Therefore, there is an urgent need for a suitable carrier to deliver encapsulated ACF to the tumor site [16].

With advancements in scientific and technological progress, chemodynamical therapy (CDT) has emerged as a viable alternative for tumor treatment in recent years [17]. CDT operates by converting intracellular H_2_O_2_ into ·OH using the iron Fenton reaction, leading to lethal toxicity specifically targeting tumor cells [18,19]. Consequently, the current research primarily concentrates on the development of nanocarriers and nano catalysts capable of transporting Fenton catalysts or metal ions [20,21,22,23]. Metal–organic frameworks (MOFs), a type of organic–inorganic hybrid material, offer considerable advantages, such as facile surface functionalization, a large specific surface area, and adjustable porosity. These properties make MOFs highly promising for applications in anticancer drug delivery [24,25]. Moreover, the numerous metal centers within MOFs can directly catalyze Fenton reactions, which has led to them becoming increasingly popular in recent years [26,27]. Cun et al. prepared an Fe_3_O_4_-based MOF (MIL-100) nanoparticle capable of generating abundant ·OH for photo-enhanced tumor therapy. The nanomaterial achieves effective anti-tumor effects by increasing ROS production. From this standpoint, CDT therapy shows promising prospects. However, the challenge remains that single CDT treatment may not fully eradicate tumors due to the hypoxic conditions within the TME [28]. Nevertheless, challenges remain in terms of poor tumor targeting and the premature elimination of MOFs from the human circulation [25]. In response, researchers have explored the use of membrane-camouflaged nanomaterials, which not only retain the inherent properties of the source cells but also exhibit excellent biocompatibility and specific targeting capabilities [29,30,31].

**Scheme 1:**
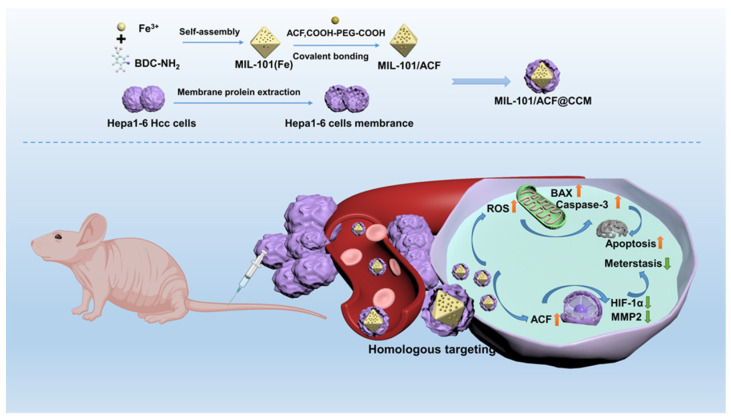
Schematic illustrations for the fabrication of MIL-101/ACF@CCM and synergistic anti-tumor effect.

In order to address the aforementioned issues, this study developed an iron-based MOF (MIL-101) as a nanocore for the delivery of ACF in combination with CDT. Differently from single CDT therapy, we combined the hypoxia-inducing factor inhibitor ACF and coated tumor cell membrane with the nanomaterial to target liver cancer cells. This novel nanomaterial is designated as MIL-101/ACF@CCM (as depicted in Scheme 1). MIL-101, which shows peroxidase activity, effectively converts the H_2_O_2_ present in the TME into ·OH, inducing oxidative stress and the subsequent apoptosis of tumor cells. Simultaneously, the loaded ACF is released at the tumor site, mitigating hypoxia in tumor cells by inhibiting the expression of HIF-1α, further enhancing the treatment of CDT. This membrane-coated MOF-based nanomaterial, characterized by its favorable biocompatibility, synergistically enhances tumor suppression through the combined therapy of ACF and CDT, thus offering a novel therapeutic approach for hepatocellular carcinoma treatment.

## 2. Materials and Methods

### 2.1. Materials

Anhydrous cobalt chloride (CoCl_2_) was obtained from Aladdin (Shanghai, China). Acriflavine (ACF) was purchased from MCE (Monmouth Junction, NJ, USA). Polyethylene glycol diacetate (COOH-PEG-COOH) was sourced from Melo PEG (Shanghai, China), and the CCK8 kit was acquired from ZOMANBIO (China, Beijing). PBS and Dulbecco’s modified Eagle’s medium (DMEM)were obtained from Gibco (Grand Island, NY, USA). Coomassie Blue Fast Staining Solution, PH fluorescent probe (BCECF AM), cell activity and cytotoxicity detection kit (CalceinAM/PI), reactive oxidative species (ROS) detection kit, and SDS-PAGE protein sample buffer (5×) were purchased from Beyotime (Shanghai, China). HIF-1α Rabbit mAb (RRID: AB_2799095), MMP-2 Rabbit mAb (RRID: AB_2799191), Bax Rabbit mAb (RRID: AB_10557411), and Caspase-3 Antibody (RRID: AB_331439) were obtained from Cell Signaling Technology (Danvers, MA, USA). N-Hydroxy succinimide (NHS) was sourced from ThermoFisher Scientific (Waltham, MA, USA). Tris-Glycine Running Buffer, Western Transfer Buffer, and 4% paraformaldehyde fixative solution were purchased from biosharp (Hefei, China). Lastly, the Cell-Light EdU Apollo567 In Vitro Kit was acquired from RIBOBIO (Guangzhou, China).

### 2.2. Methods

#### 2.2.1. Preparation of MIL-101/ACF

The synthesis of MIL-101 (Fe) nanoparticles involved a solvent heat treatment method. In this process, 30 mg of MIL-101 (Fe) and 60 mg of COOH-PEG-COOH were dispersed in 30 mL of ultra-pure water. To facilitate the reaction, 30 mg of NHS and 50 mg of EDC were added. The mixture was then subjected to ultrasonication in a water bath for 2 h, followed by overnight stirring in the absence of light. Afterward, the resulting nanoparticles were washed with anhydrous ethanol and dried for further use. Next, ACF molecules were covalently attached to the MIL-101 (Fe) nanoparticles through an amide reaction. The specific steps involved dissolving 60 mg of ACF in a 30 mL solution of anhydrous ethanol. Subsequently, 30 mg of MIL-101 (Fe) coated with PEG was dispersed in the ACF solution and stirred away from light for a duration of 24 h. The resulting mixture was then washed with anhydrous ethanol and subsequently dried through centrifugation, resulting in the formation of MIL-101/ACF nanoparticles.

#### 2.2.2. Preparation of Homologous Liver Cancer Cell Membrane (CCM)

Initially, hepatocellular carcinoma cells (Hepa1-6) were cultured up to approximately 6 × 10^7^ cells/cm^2^. Subsequently, the cells were detached and subjected to centrifugation to obtain cellular particles. The cells were then subjected to treatment with membrane protein extraction reagent A (Membrane and Cytosol Protein Extraction Kit come from Beyotime) containing Phenylmethanesulfonyl fluoride (PMSF). Following this, the cells were suspended in ice for a duration of 15 min and subsequently transferred to a glass homogenizer that had been pre-cooled in an ice bath. Homogenization was performed approximately 60 times. After the homogenization process, centrifugation was carried out at 4 °C and 700× *g* for a period of 10 min in order to eliminate the nucleus and intact cells. The resulting supernatant was further subjected to centrifugation at 4 °C and 14,000× *g* for 30 min to collect the cell membrane. Following the removal of the supernatant, the cell membrane was collected, freeze-dried, and stored for future use.

#### 2.2.3. Preparation of MIL-101/ACF@CCM Nanoparticles

The cell membrane was coated using the ultrasonic method. A suitable quantity of MIL-101/ACF and the extracted cell membrane were dispersed in 1 mL of PBS and placed in the ultrasonic cell crusher. Next, the prepared MIL-101/ACF is mixed with the cell membrane was then positioned under an ice bath for a duration of 10 min, with a power output of 300 W, operating for 3 s and pausing for 2 s in an intermittent manner. Subsequently, the coated nanoparticles were obtained by repeatedly washing the sample with water to eliminate any residual blank cell membrane vesicles, resulting in the formation of MIL-101/ACF@CCM nanoparticles with a membrane coating.

#### 2.2.4. Characterization of MIL-101/ACF@CCM Nanoparticles

The copper mesh, which is suitable for electron microscope analysis, was positioned on a pristine filter paper. Subsequently, 10 μL of MIL-101, MIL-101/ACF, and MIL-101/ACF@CCM solution were absorbed onto the copper mesh using a pipetting gun. The excess solution was soaked up by the filter paper, followed by the addition of a 1% phospho-tungsten acid solution for staining. The assembly was then placed in an oven at a temperature of 37 °C overnight. The morphology of the sample was examined using a transmission electron microscope (TEM). To determine the particle size and Zeta potential, the prepared MIL-101, MIL-101/ACF, and MIL-101/ACF@CCM samples were diluted with double-distilled water and placed in a specialized colorimetric plate for analysis using a Malvern dynamic light-scattering analyzer.

#### 2.2.5. Characterization of Membrane Proteins via SDS-Gel Electrophoresis

The protein concentrations of Hepa1-6 cells, extracted Hepa1-6 cell membrane, and MIL-101/ACF@CCM were determined using a BCA kit. Subsequently, the sample was diluted with SDS-PAGE protein loading buffer to ensure uniform concentration based on the detected protein concentration. The diluted sample was then subjected to boiling and cooling in a 95 °C water bath for future use. SDS-PAGE gel electrophoresis was performed to obtain the gel, which was subsequently stained with Coomassie Blue Fast Staining Solution for 1 h. The stained gel was repeatedly washed with a decolorizing solution until the band became clearly visible.

#### 2.2.6. Hydroxyl Radical Detection via UV-Vis

The detection of the catalytic activity of MIL-101/ACF@CCM towards H_2_O_2_ to ·OH was performed by utilizing 3,3′,5,5′-tetramethylbenzidine (TMB) as the substrate. The production of ·OH was evaluated under varying concentrations of nanomaterials, incubation times, TMB concentrations, and H_2_O_2_ concentrations. The test solution was analyzed using an ultraviolet spectrophotometer to record the ultraviolet–visible absorption spectra at 650 nm.

#### 2.2.7. ACF Drug Load Calculation

The experiment was conducted using ultraviolet spectrophotometry to establish a linear regression model by measuring the absorbance of ACF at various concentrations and collate the tested data. The absorption rate of ACF was determined at 460nm, enabling calculation of the drug concentration using the following formula. DLC = drug loading content; DLE = drug loading efficiency.

$$DLC=\frac{Quantity\;of\;ACF\;in\;raw\;materials-Quantity\;of\;ACF\;in\;supernatant}{amount\;of\;loaded\;(MIL-101+ACF)}\times 100\%$$

$$DLE=\frac{Quantity\;of\;ACF\;in\;raw\;materials-Quantity\;of\;ACF\;in\;supernatant}{Quantity\;of\;ACF\;in\;raw\;materials}\times 100\%$$

#### 2.2.8. Cell Culture

For the in vivo and in vitro experiments, mouse hepatocellular carcinoma Hepa1-6 cells (Hepa1-6), human normal hepatic epithelial cells THLE-2, and Hepa1-6-LUC cells were utilized. All cells were cultivated in a humidity-controlled incubator at 37 °C with a partial pressure of 5% CO_2_. The modified medium DMEM, containing 10% fetal bovine serum, 1% penicillin/streptomycin, and 200 μM CoCl_2_, was used. It is important to note that the cells used for the subsequent in vitro experiments were cultured under hypoxic conditions (the hypoxic conditions are provided by 200 μM CoCl_2_).

#### 2.2.9. Cell Uptake Assay

The internalization of MIL-101/ACF@CCM into Hepa1-6 cells was observed using an inverted fluorescence microscope. Hepa1-6 cells were digested and then seeded into 24-well plates at a density of 2 × 10^4^ cells per well. The plates were then incubated overnight in a cell incubator at 37 °C and 5% CO_2_ until the cells adhered to the surface of the well. MIL-101/ACF@CCM was introduced to the cells, resulting in a final ACF concentration of 2.5 μg/mL. The cells were subsequently incubated for 0, 2, 4, and 8 h. Following the incubation period, the cells were washed, fixed, and stained with DAPI. After a PBS wash, the uptake of MIL-101/ACF@CCM was assessed using an inverted fluorescence microscope.

#### 2.2.10. Cytotoxicity Test

The cytotoxic effects of MIL-101, MIL-101/ACF, and MIL-101/ACF@CCM were assessed using the CCK8 method. For this experiment, Hepa1-6 and THLE-2 cells were cultured in 96-well plates at a density of 5 × 10^4^ cells per well, with a volume of 100 μL per well. Various concentrations of MIL-101, MIL-101/ACF, and MIL-101/ACF@CCM (ranging from 0 to 350 μg/mL) were added to the cells and incubated for 24 h. The cell viability of each group was then determined using the CCK8 method at a wavelength of 450 nm.

#### 2.2.11. In Vitro ROS Test

DCFH-DA was utilized as a fluorescent probe to detect intracellular ROS. Hepa1-6 cells were cultured in a 24-well plate, and 500 μL of the cells were treated with various drugs and concentrations. The groups included the following: Control; MIL-101 at 300 μg/mL; ACF at 2.5 μg/mL; MIL-101/ACF at 300 μg/mL; MIL-101/ACF@CCM at 300 μg/mL. The subsequent in vitro experiments were conducted by incubating the cells with the aforementioned groups and concentrations for a period of 12 h. Following the incubation, DCFH-DA reagent was added in an appropriate quantity and the resulting fluorescence was observed under an inverted fluorescence microscope after a 15 min incubation. The Statistical fluorescence value was analyzed using Image J 1.x.

#### 2.2.12. Calcein-AM/PI Test

The Hepa1-6 cells were introduced into 6-well plates at a density of 6 × 10^5^ cells per well. Once the cells adhered to the surface, various drugs were administered to treat them. Following a 24 h incubation period, 1 μL of Calcein-AM and 2 μL of PI were sequentially added and the cells were further cultured for a specific duration. Fluorescence images were captured after staining, and the cell count was determined using Image J 1.x software.

#### 2.2.13. EdU Cell Proliferation Experiment

The Hepa1-6 cells were introduced into 48-well plates at a density of 2 × 10^4^ cells per well. Following cell adhesion, various drugs were administered and the cells were incubated for 24 h. Subsequently, the EdU kit guidelines were followed to label the cells, which were then fixed with a 4% solution of paraformaldehyde. EdU reagent and DAPI nucleation were added to the cells, and images were captured using an inverted fluorescence microscope. The number of cells was quantified using Image J 1.x software.

#### 2.2.14. Colony-Formation Assay

Hepa1-6 cells were introduced into 6-well plates at a concentration of 1 × 10^3^ cells per well. Following adhesion, the cells were subjected to various drug treatments and cultured until the formation of colonies, with the medium being replaced every 3 days. Once colonies had formed, they were fixed with 4% paraformaldehyde for a duration of 30 min. Subsequently, 1 mL of 0.1% crystal violet was added and allowed to incubate for 30 min. After thorough washing, the cells were dried and captured in images. The cell count was determined using Image J 1.x software.

#### 2.2.15. Cell Immunofluorescence

Hepa1-6 cells were seeded at a density of 3 × 10^5^ cells per well in a 24-well plate coated with coverslip. After the cells adhered to the plate, they were treated with various concentrations of drugs and incubated for 12 h. The medium was then removed, and the cells were fixed with 4% paraformaldehyde for 15 min. Following PBS cleaning, 0.5% TritonX-100 PBS solution was added and incubated at room temperature for 20 min. Subsequently, the fast-sealing solution was applied for 15 min, followed by overnight incubation with the primary antibody (dilution, 1:200). The cells were then stained with Cy3 (dilution, 1:500) secondary antibody for 1 h in order to shield the light. Finally, DAPI was added for 10 min to stain the nuclei. An anti-quenching agent was applied to the coverslip, and, after sealing the coverslip, images were captured using a confocal microscope. The fluorescence intensity was measured using Image J 1.x.

#### 2.2.16. Intracellular pH Determination

The intracellular pH was measured by employing BCECF-AM as a fluorescent indicator in this study. Hepa1-6 cells were seeded into 24-well plates at a density of 3 × 10^5^ cells per well. Once the cells adhered to the plate, various drugs were introduced and allowed to incubate for a period of 24 h. Subsequently, BCECF-AM (5μM) was applied and allowed to stain the cells in darkness for 30 min, followed by three washes with PBS. Fluorescence images were captured using an inverted fluorescence microscope, and the fluorescence values were determined using Image J 1.x software.

#### 2.2.17. Wound Healing

To initiate the experiment, Hepa1-6 cells were cultured on 6-well plates until they reached a fusion rate of 90%. Subsequently, a scratch wound was deliberately inflicted using the tip of a 200 μL pipette. The wound area was carefully washed twice with PBS to eliminate any debris. Following this, a serum-free medium containing distinct nanoparticles was introduced, and the cells were subjected to a low-oxygen environment (the hypoxic conditions are provided by 200 μM CoCl_2_) for a duration of 24 h. Microscopic images were captured before and after the 24 h incubation period, and the extent of wound closure was assessed using the semi-quantitative software, Image J 1.x.

#### 2.2.18. Migration Assay

The Hepa1-6 cells were cultured in serum-free DMEM medium for a duration of 12 h. Subsequently, the cells were digested and introduced into the upper chamber of a transwell (24 well). The upper chamber contained 100 μL of medium with 10% fetal bovine serum, while the lower chamber contained 600 μL of medium with 20% fetal bovine serum. Once the cells attached to the wall, they were subjected to various drug treatments and incubated for a period of 24 h. Following this, the cells were fixed with 4% paraformaldehyde, stained with 0.1% crystal violet for a duration of 30 min, and then dried. The cells were observed under an inverted microscope, and the cell number was analyzed using Image J 1.x software.

#### 2.2.19. Western Blot

The protein was obtained from various treated tumor tissues through the process of grinding. The protein concentration was standardized using the BCA quantitative method. Subsequently, the expression level of HIF-1α and other proteins (dilution, 1:1000) was assessed using denaturing polyacrylamide gel electrophoresis.

#### 2.2.20. In Vivo Distribution Study

This study employed IR780 as a fluorescent probe to label MIL-101/ACF and MIL-101/ACF@CCM for the purpose of imaging small animals in vivo. To begin, we employed ultrasonic mixing to combine IR780 and MIL-101/ACF in a 1:4 ratio. The mixture was then stirred overnight in a dark environment. After several rounds of washing and centrifugation, the unbound IR780 was separated through centrifugation, resulting in the successful production of IR780-labeled MIL-101/ACF. Subsequently, a cancer cell membrane was applied to coat the IR780-labeled MIL-101/ACF, resulting in the formation of IR780-labeled MIL-101/ACF@CCM. The investigation focused on the distribution of MIL-101/ACF and MIL-101/ACF@CCM nanoparticles in mice with Hepa1-6 tumor. Once the tumor volume reached approximately 150 mm^3^, MIL-101/ACF and MIL-101/ACF@CCM nanoparticles were separately injected into the tail vein at a dosage of 3 mg/kg. The mice were anesthetized at intervals of 0, 2, 4, 6, 8, 10, and 12 h after injection, and the distribution of nanoparticles in tumor-bearing mice was observed by using IVIS spectrum of small animal imager. Fluorescence imaging was selected, and the excitation light was set at 780 nm for fluorescence observation. Following this, the mice were euthanized via neck dislocation, and the heart, liver, spleen, lung, kidney, and tumor tissues were collected. The distribution of the nanoparticles in each major organ was observed using a small-animal living imager.

#### 2.2.21. Tumor Modeling and In Vivo Antitumor Effect

In this experiment, 30 healthy 4-week-old female BALB/c nude mice with a body weight of about 18~20 g were selected and fed in an SPF environment with a temperature of 23 °C and relative humidity of 50% to establish a tumor-bearing nude mouse model. They were randomly divided into 5 groups, and each group had 6 animals. The present study was approved by the Ethics Committee of Affiliated Hospital of Guilin Medical University. The groups were classified as follows: Control group receiving PBS; MIL-101 group receiving a dosage of 3 mg/kg; ACF group receiving a dosage of 25 μg/kg; MIL-101/ACF group receiving a dosage of 3 mg/kg; and MIL-101/ACF@CCM group receiving a dosage of 3 mg/kg. The drug was administered through intravenous injection every four days. Subsequently, the tumor volume and body weight of the mice were recorded following the treatment period. After 12 days, all the mice were humanely euthanized, and blood samples were collected to determine various biochemical indexes. Furthermore, heart, liver, spleen, lung, kidney, and tumor samples were collected for HE staining and subsequent histological analysis.

#### 2.2.22. Statistical Analysis

The experimental data were subjected to analysis using GraphPad one-way analysis of variance (ANOVA) implemented in Prism8.3 software. The calculated probabilities (*P*) were denoted as follows: * for *p* < 0.05, ** for 0.001 < *p* < 0.01, and *** for *p* < 0.001. The abbreviation “ns” was used to indicate that the observed differences were not statistically significant.

## 3. Results

### 3.1. Synthesis and Characterization of MIL-101/ACF@CCM

**Figure 1:**
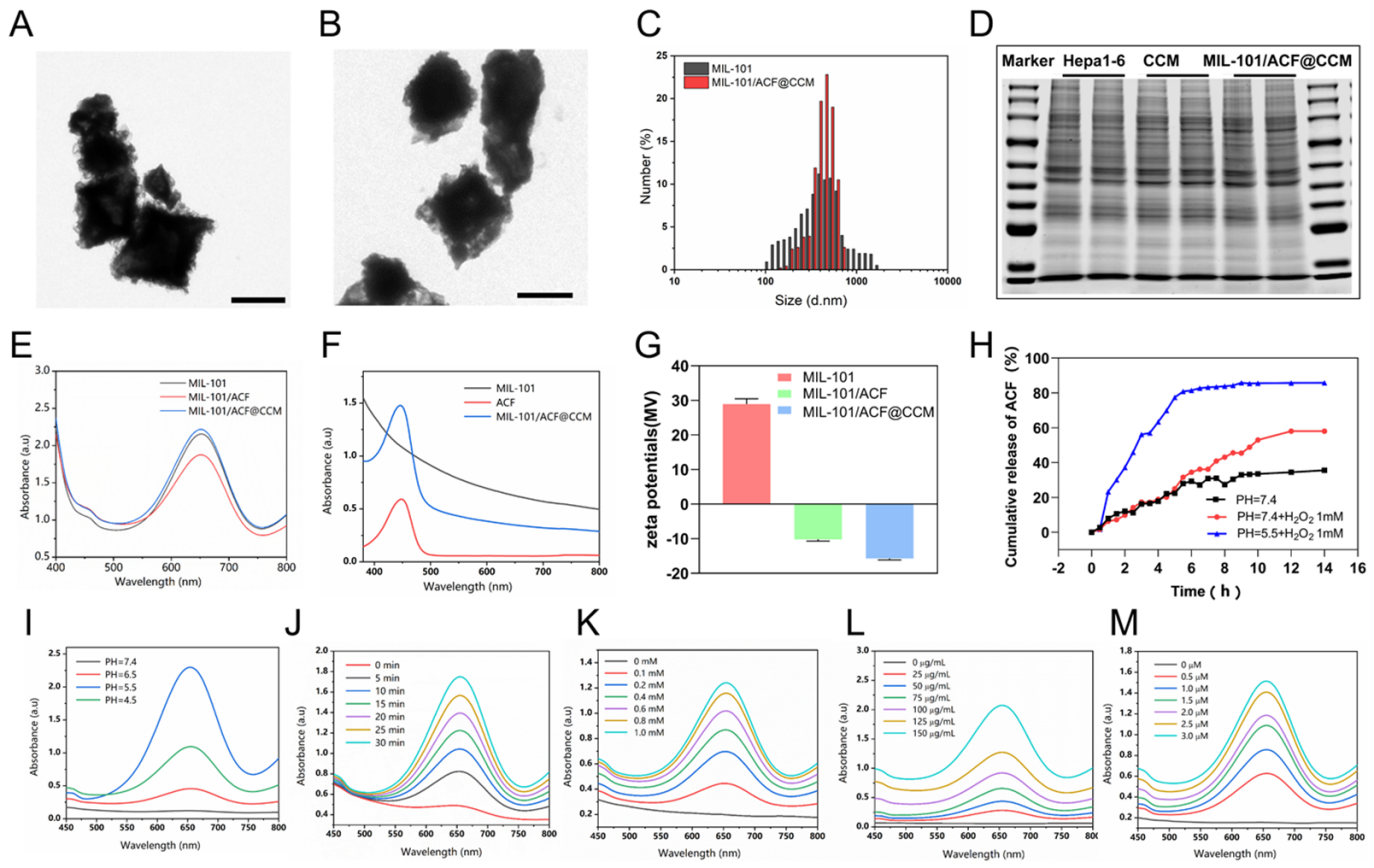
Characterization of MIL-101/ACF@CCM. TEM images of MIL-101 (**A**) and MIL-101/ACF@CCM (**B**), scale bar: 500 nm. (**C**) DLS of MIL-101 and MIL-101/ACF@CCM. (**D**) SDS-PAGE rapid staining and decolorization. (**E**) UV-vis detecting the TMB. (**F**) UV-vis detecting the drug loading of MIL-101/ACF@CCM. (**G**) The Zeta potential of MIL-101, ACF, MIL-101/ACF, and MIL-101/ACF@CCM. (**H**) Release profile of ACF from MIL-101/ACF@CCM at different H_2_O_2_ concentration and phosphate buffer. (**I**) UV-vis absorption spectra of MIL-101/ACF@CCM in the sodium acetate buffer solution (different pH). (**J**) Time-dependent UV-vis absorption spectra of MIL-101/ACF@CCM (150 μg·mL^−1^) (in the pH = 5.5 of the sodium acetate buffer solution). UV-vis absorption spectra of MIL-101/ACF@CCM in the sodium acetate buffer solution (pH = 5.5). (**K**) Different concentrations of TMB. (**L**) Different concentrations of MIL-101/ACF@CCM. (**M**) Different concentrations of H_2_O_2_.

The methodology for the preparation of MIL-101/ACF@CCM is illustrated in Scheme 1. Initially, MIL-101 (Fe) was synthesized via the thermal flux method, involving the self-assembly of iron and 2-amino-terephthalic acid trimers [32]. MIL-101 (Fe) exhibits versatile applications in drug carriers, adsorption materials, and catalysis. To enhance the binding stability between MIL-101 and ACF molecules, a layer of PEG with a carboxyl group was synthesized and immobilized on the exterior of MIL-101. This approach aimed to achieve the covalent binding of MIL-101 and ACF molecules through an amide reaction [33]. The drug loading rate of MIL-101 was determined using UV-visible spectroscopy. Notably, ACF exhibited a characteristic absorption peak at 460 nm (Figure S1). To establish a standard curve for ACF, its characteristic peak at 460 nm was utilized (Figure S2). The calculated drug loading rate was approximately 48.7%, and the encapsulation rate was 73%, indicating the favorable drug loading capacity of MIL-101. Subsequently, the nanoparticles were coated with a cancer cell membrane using the ultrasound method [34,35], resulting in the designation of MIL-101/ACF@CCM. The morphology characteristics of MIL-101 and MIL-101/ACF@CCM were examined using TEM. According to the depicted Figure, MIL-101 exhibited an octahedral structure with an average diameter of approximately 400 nm (Figure 1A). Additionally, the TEM images revealed that MIL-101/ACF@CCM was encapsulated by a cell membrane (Figure 1B). The analysis conducted using dynamic light scattering (DLS) confirmed the diameter of MIL-101 to be around 400 nm. After being wrapped in the cell membrane, the diameter of MIL-101/ACF@CCM increased to approximately 450 nm, providing further evidence of successful membrane encapsulation (Figure 1C). To analyze the proteins present in Hepa1-6 cells, Hepa1-6 cell membranes, and MIL-101/ACF@CCM samples, the SDS-PAGE rapid staining and decoloration method was employed. The outcomes, showcased in Figure 1D, demonstrated that MIL-101/ACF@CCM exhibited a protein band distribution consistent with that of Hepa1-6 cell membrane samples, indicating the successful retention of major membrane proteins by the nanoparticle. In order to comprehensively characterize the properties of the prepared MIL-101/ACF@CCM bionic nanoparticles, UV-vis spectroscopy was utilized to investigate whether the catalytic ability of MIL-101 to produce ·OH changed after drug loading and film coating. As depicted in Figure 1E, the results indicated no alteration in its properties. Furthermore, the presence of the characteristic peak of ACF at 460 nm was utilized to further confirm the successful loading of ACF (Figure 1F). The synthesis of MIL-101/ACF@CCM was confirmed by the Zeta potential (Figure 1G). The release of ACF from MIL-101/ACF@CCM was investigated in various solutions including PBS, hydrogen peroxide, and acid hydrogen peroxide. The results, as depicted in Figure 1H, indicate that ACF was released from MIL-101/ACF@CCM over a period of 10 h. Within the first 10 h of soaking in an acidic hydrogen peroxide solution (1 mM), approximately 85.8% of ACF was released. However, when soaked with hydrogen peroxide or PBS, the release of ACF was reduced to 58% and 35%, respectively. Subsequently, the ability of MIL-101/ACF@CCM to catalyze the generation of ·OH by H_2_O_2_ was examined. This investigation, as shown in Figure 1I–M, demonstrated that the catalytic ability of MIL-101/ACF@CCM increased with the concentrations of TMB and H_2_O_2_, as well as the incubation time of the nanomaterial. Additionally, it was observed that MIL-101/ACF@CCM exhibited enhanced catalytic performance under weakly acidic conditions (pH = 5.5) in different sodium acetate solutions of varying pH (Figure 1I). This finding suggests that MIL-101/ACF@CCM could potentially play a more effective role in tumor microenvironments characterized by weak acidity.

### 3.2. Cell Uptake and Cytotoxicity of MIL-101/ACF@CCM In Vitro

**Figure 2:**
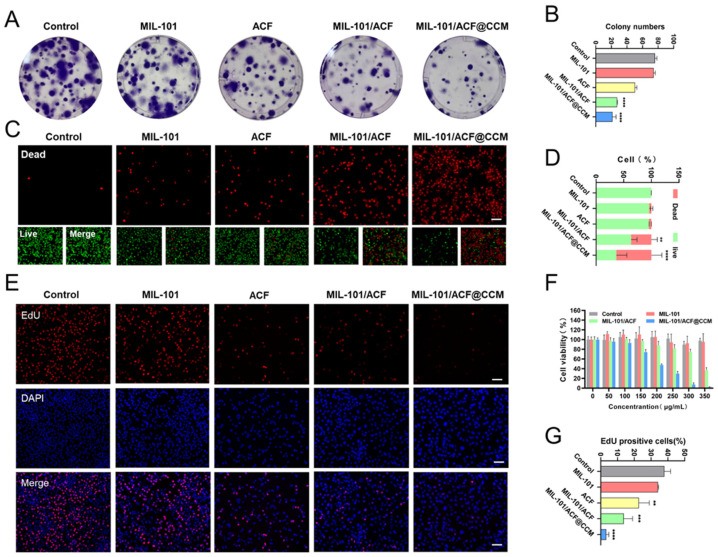
Cell uptake and cytotoxicity of MIL-101/ACF@CCM in vitro. (**A**) The clone images show Hepa1-6 cells incubated under hypoxic conditions and the corresponding number of colonies measured by Image J (**B**) under different treatments. (**C**) Images of hypoxic Hepa1-6 cells after incubation with Calcein/PI and the corresponding fluorescence intensities measured by Image J (**D**) under different treatments. (**E**) Images of hypoxic Hepa1-6 cells after incubation with EdU/DAPI and the corresponding EdU-positive cells measured by Image J (**G**) under different treatments. (**F**) Cell viability of Hepa1-6 cells incubated with different concentrations of material (MIL-101, MIL-101/ACF, MIL-101/ACF@CCM). Scale bar: 50 μm. n = 3, ** *p* < 0.01, *** *p* < 0.001, **** *p* < 0.0001.

Following the successful synthesis of MIL-101/ACF@CCM and its demonstrated ability to catalyze H_2_O_2_ production of ·OH, the mouse hepatoma cell line Hepa1-6 was employed as a model for tumor cells. To simulate the anoxic microenvironment of tumors, CoCl_2_ was utilized. Initially, a concentration of 200 μM of CoCl_2_ was chosen to establish an anoxic cell model through the CCK8 method and Western blot assay (Figures S3 and S4). This anoxic cell model was subsequently employed in further cell experiments. The uptake capacity of MIL-101/ACF@CCM by Hepa1-6 cancer cells was observed using an inverted fluorescence microscope. As the co-culture time between Hepa1-6 cancer cells and MIL-101/ACF@CCM increased, the fluorescence signal of ACF detected in the cancer cells grew stronger, indicating the successful ingestion of MIL-101/ACF@CCM (Figure S5). In order to assess the tumor-specific cytotoxicity of MIL-101/ACF@CCM nanoparticles, various drugs were co-incubated with tumor cells in vitro, and the CCK8 kit was employed to measure drug toxicity. It was observed that the MIL-101/ACF@CCM group exhibited greater cytotoxicity compared to both the control group and the MIL-101/ACF group (Figure 2F). However, in the normal hepatic epithelial cells THLE-2, the MIL-101/ACF@CCM group did not demonstrate significant effects on the cells (Figure S6), suggesting a certain level of biosafety associated with MIL-101/ACF@CCM. To further investigate the anti-tumor efficacy of the MIL-101/ACF@CCM administration group, the colony-forming ability of cells was evaluated through a cloning experiment. The results depicted in Figure 2A,B demonstrate the evident cell inhibition ability of the MIL-101/ACF@CCM treatment group. Additionally, the apoptosis of cells in each drug administration group was examined using living and dead staining, and the proportion of living to dead cells was analyzed. Figure 2C,D reveal a significant increase in the apoptosis rate induced by MIL-101/ACF@CCM in the administration group. Furthermore, the cell proliferation experiment (Figure 2E) substantiated the notable anti-tumor effects of the administration group MIL-101/ACF@CCM. Statistical analysis using Image J semi-quantification also yielded statistically significant results (Figure 2G).

### 3.3. Functional Experiment Using MIL-101/ACF@CCM In Vitro

**Figure 3:**
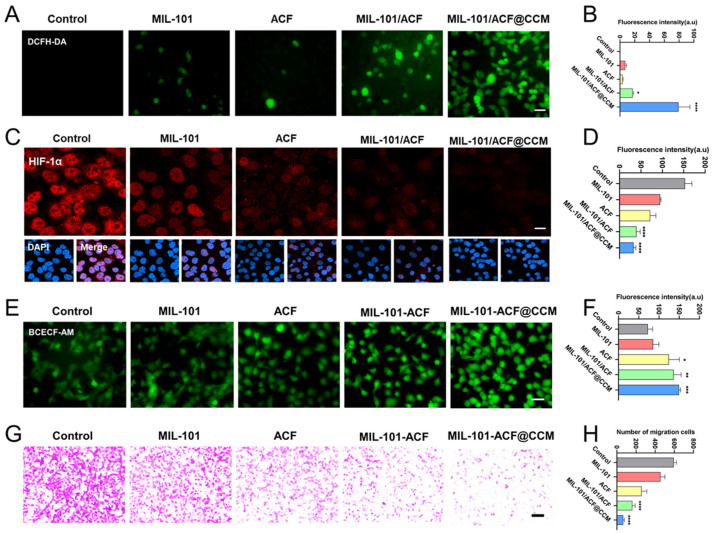
Functional experiment using MIL-101/ACF@CCM in vitro. (**A**) Images of ROS generation of Hepa1-6 cells under incubated hypoxic condition and the corresponding fluorescence intensities measured by Image J (**B**) under different treatments. (**C**) CLSM images (HIF-1α antibody and DAPI staining) of hypoxic Hepa1-6 cells and the corresponding fluorescence intensities measured by Image J (**D**) under different treatments. (**E**) Images of hypoxic Hepa1-6 cells after incubation with BCECF-AM to indicate the intracellular pH value and the corresponding fluorescence intensities measured by Image J (**F**) under different treatments. (**G**) Transwell images of Hepa1-6 cells incubated under hypoxic condition values and the corresponding number of migration cells measured by Image J (**H**) under different treatments. Scale bar: 50 μm. n = 3, * *p* < 0.05, ** *p* < 0.01, *** *p* < 0.001, **** *p* < 0.0001.

The strong anti-tumor ability of the MIL-101/ACF@CCM group was hypothesized to be attributed to the increased production of ROS and the inhibition of HIF-1α expression through the release of ACF. To test this hypothesis, the intracellular ROS levels were initially assessed by observing the fluorescence intensity of the ROS probe DCFH-DA using inverted fluorescence microscopy. The results, as depicted in Figure 3A,B, demonstrated that the MIL-101/ACF@CCM group exhibited the highest ROS fluorescence, while the production of intracellular ROS by MIL-101 and ACF alone was limited. Subsequently, the ability of ACF release to inhibit tumor cell migration was evaluated through cell immunofluorescence experiments, which revealed that MIL-101/ACF@CCM effectively suppressed HIF-1α expression (Figure 3C). Statistical analysis further confirmed a significant difference between the control group and the MIL-101/ACF@CCM group (Figure 3D). Furthermore, the inhibition of HIF-1α expression was believed to ameliorate the hypoxic conditions within the tumor microenvironment [36]. Consequently, the intracellular acidic pH was examined using the pH probe BCECF-AM to investigate whether MIL-101/ACF@CCM could improve the acidic environment in tumor cells (Figure 3E,F), thereby indicating an alleviation of hypoxia. As anticipated, the results demonstrated that MIL-101/ACF@CCM effectively improved the acidic environment within tumor cells. Research has demonstrated that the mitigation of hypoxia has a partial inhibitory effect on the metastasis of tumor cells [37]. Consequently, we conducted further investigations to examine how the release of ACF from MIL-101/ACF@CCM impacts the migration of tumor cells. Experimental transwell findings indicate that MIL-101/ACF@CCM significantly decreases the migration of Hepa1-6 cells (Figure 3G,H). Additionally, we assessed the impact of MIL-101/ACF@CCM on the healing rate of Hepa1-6 cells using a scratch experiment. After 24 h of incubation under hypoxia, the control group exhibited a noteworthy reduction in the size of the scratch area, indicating that Hepa1-6 cells have a robust wound healing capability. However, the application of MIL-101/ACF@CCM effectively inhibited the healing of the wound (Figures S7 and S8).

### 3.4. Antitumor Effects of MIL-101/CCM In Vivo

**Figure 4:**
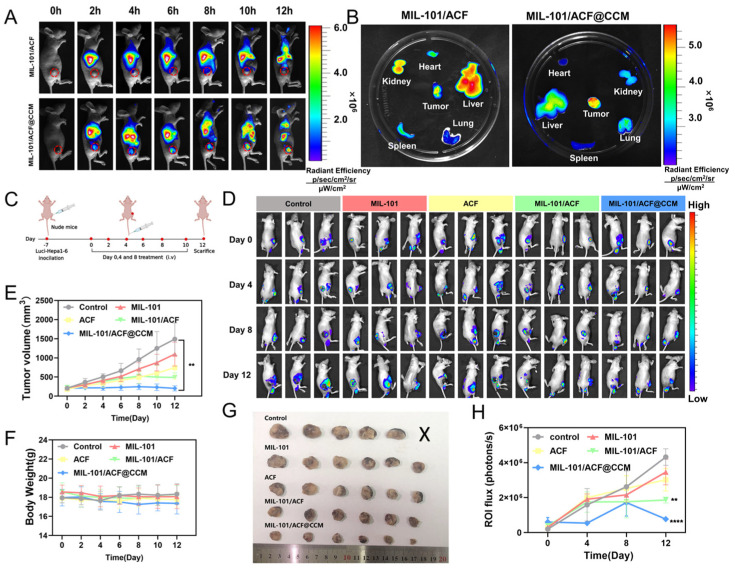
Antitumor of MIL-101/ACF@CCM in vivo. (**A**) In vivo fluorescence imaging of mice with intravenous injection of MIL-101/ACF or MIL-101/ACF@CCM. (**B**) Ex vivo fluorescence images of the tumor tissues and other major organs harvested from Hepa1-6 tumor-bearing mice after 24 h post intravenous administration of IR780-labeled MIL-101/ACF and MIL-101/ACF@CCM. (**C**) Time axis of in vivo anti-tumor experiments and operation details. (**D**) Bioluminescence imaging for intuitive tumor surveillance in mice at different periods. (**E**) Relative tumor volume changes in tumor tissues in the whole process after various treatments. (**F**) The changes in the mouse relative weight through the whole process after various treatments. (**G**) The photographs of excised tumors at the end of treatment after various treatments. (**H**) The quantitate luminescence of the ROI from Figure 4D. n = 6, ** *p* < 0.01, **** *p* < 0.0001.

**Figure 5:**
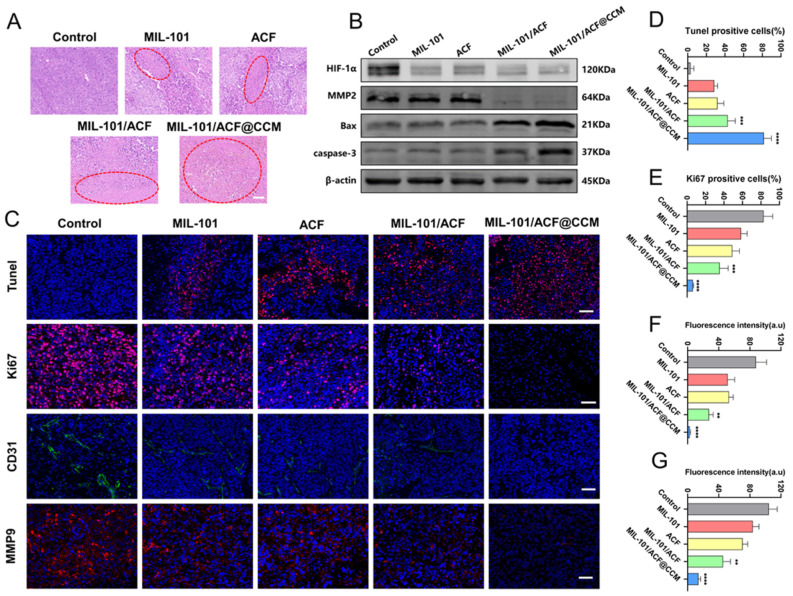
Antitumor effects of MIL-101/ACF@CCM in vivo. (**A**) H&E staining analysis of tumor tissues at 12 days after Hepa1-6-LUC cell inoculation in different groups. The area circled in red represents the region of cell damage. (**B**) Western blot bands of various proteins collected from Hepa1-6 tumors implanted on tumor-bearing mice that experienced corresponding treatments in different treatment groups. (**C**) Tunel, Ki-67, CD31, and MMP9 staining of tumor tissue from various treatments. (**D**–**G**): The relevant fluorescence quantification of Tunel, Ki67, CD31, and MMP9. Scale bar: 50 μm. n = 3, ** *p* < 0.01, *** *p* < 0.001, **** *p* < 0.0001.

In vitro experiments have demonstrated the favorable characteristics of MIL-101/ACF@CCM, such as effective cell targeting, efficient cellular uptake, and the induction of apoptosis. These findings have prompted us to further investigate the potential anti-tumor of MIL-101/ACF@CCM in vivo. To assess the biosafety of MIL-101/ACF@CCM, a solution containing MIL-101/ACF@CCM at a concentration of 200 μg/mL was injected into healthy mice via the tail vein. Blood biochemical indicators were then measured 24 h after injection. The results revealed that these indicators fell within the normal range (Figure S9), suggesting that MIL-101/ACF@CCM can be safely administered to mice with tumors. Moreover, different concentrations of MIL-101/ACF@CCM were incubated with red blood cells at 37 °C for 4 h. In vitro hemolysis experiments confirmed the favorable biosafety profile of MIL-101/ACF@CCM (Figure S10). As a result, we established a model using Hepa1-6-Luc that stably expressed luciferase to assess the in vivo distribution and anti-cancer effects of MIL-101/ACF@CCM. To evaluate tumor accumulation, MIL-101/ACF and MIL-101/ACF@CCM were labeled with IR780, and their accumulation in tumors was assessed using an in vivo imaging apparatus for small animals. Figure 4A depicts the gradual enhancement of fluorescence signals at the tumor site following caudal vein injection after two hours. The MIL-101/ACF@CCM group exhibited greater tumor enrichment compared to the MIL-101/ACF group, with the maximum accumulation time being approximately 12 h. These findings suggest that homologous cell membranes offer superior tumor-targeting capabilities. After twenty-four hours of injection, the mice were euthanized, and the major organs (heart, liver, spleen, lung, kidney) and tumors were collected for the analysis of their in vitro biological distribution. Although MIL-101/ACF@CCM exhibited strong liver metabolism, it also showed a relatively high rate of accumulation in the tumor region, which met the basic treatment requirements (Figure 4B), and Figure S11 show the quantitation of fluorescence from each organ ex vivo. Consequently, therapeutic drugs were injected into the tail vein and the mice were euthanized after three treatments for further analysis (Figure 4C). The tumor growth was monitored at different time intervals using fluorescence imaging (Figure 4D,H), while the tumor volume and mouse weight were recorded every two days. As anticipated, mice treated with MIL-101/ACF@CCM displayed the slowest tumor growth throughout the treatment period, with noticeable differences in tumor volume on the final day when compared to the control group (Figure 4E), and no significant change in body weight (Figure 4F). The images and tumor weight of the tumors removed after various treatments are presented in Figure 4G and Figure S12. The tumor growth in the MIL-101/ACF@CCM group was observed to be slower than that in the control group. H&E staining revealed that the cell damage (the area circled in red) in the MIL-101/ACF@CCM group was the most severe compared to the control group and the MIL-101/ACF group (Figure 5A). Furthermore, due to the commendable catalytic ability of MIL-101/ACF@CCM and its ability to inhibit HIF-1α, our investigation focused on determining whether the generation of ·OH and the release of ACF by MIL-101/ACF@CCM can impede tumor growth through apoptosis induction and the inhibition of HIF-1α expression. To assess tumor inhibition following MIL-101/ACF@CCM treatment, Western blotting and Ki67 staining were employed. The findings depicted in Figure 5B demonstrate that post treatment, the MIL-101/ACF@CCM treatment group exhibited elevated levels of the apoptosis-promoting proteins Bax and Caspase-3, while the expressions of HIF-1α and MMP2, both involved in cell migration, decreased. These results signify the effective suppression of tumor proliferation by MIL-101/ACF@CCM. Consistent outcomes were also observed through tumor tissue staining (Figure 5C); the relevant fluorescence quantification is shown in Figure 5D–G.

## 4. Discussion

In summary, the MOF metal framework MIL-101 (Fe) was chemically bonded to the HIF-1α inhibitor ACF and coated with the plasma membrane of tumor cells to synthesize the MOF nanoparticles MIL-101/ACF@CCM. These nanoparticles exhibit the capability to form H_2_O_2_ and generate ·OH through the Fenton reaction, specifically in response to the acidic tumor microenvironment. Moreover, they possess the ability to enhance drug accumulation at tumor sites by exploiting the homologous targeting of cancer cell membranes, solving the problem of poor targeting of MOF materials. On one hand, the tumor-specific CDT facilitated by MIL-101/ACF@CCM can lead to increased production of ROS at the tumor site, thereby inducing apoptosis in tumor cells and inhibiting their growth. On the other hand, MIL-101/ACF@CCM effectively suppresses the expression of HIF-1α, thereby improving the hypoxic conditions of the tumor microenvironment and further making up for the deficiency of single CDT therapy. In vitro and in vivo experiments have demonstrated the excellent biocompatibility and potent anti-tumor and anti-metastasis effects of MIL-101/ACF@CCM. At present, metal-based nanomaterials can enhance metal immunotherapy through unique biological effects [38]. The iron-based nanomaterials studied in our study have been proven to be able to target to tumor sites. In future studies, we hope to further explore whether MIL-101/ACF@CCM can release iron ions at tumor sites to activate tumor immunotherapy. In summary, this research work offers a promising therapeutic strategy for the treatment of hepatocellular carcinoma, which not only reduces adverse effects but also presents a novel approach to tumor-specific treatment.

## Data Availability

Data are contained within the article and Supplementary Materials.

## Supplementary Materials

The following supporting information can be downloaded at: https://www.mdpi.com/article/10.3390/pharmaceutics16050619/s1, Figure S1: UV-vis absorption spectra of ACF in H_2_O solution at different concentrations; Figure S2: The linearly fitted standard curve of ACF characteristic absorption intensity at 460 nm vs. concentration; Figure S3: Western blot bands of hypoxia-related proteins (i.e., HIF-1α) in Hepa1-6 cells after incubating with CoCl_2_ with varied concentrations; Figure S4: Cell viability of Hepa1-6 cells after incubating with CoCl_2_ with varied concentrations; Figure S5: Cell internalization of MIL-101/ACF@CCM nanoagents by Hepa1-6 cells; Figure S6: Cell viabilities of THLE-2 cells after incubation with varied concentrations of MIL-101/ACF@CCM nanoagents for 24 h; Figure S7: Images of wound healing of hypoxic Hepa1-6 cells after different treatments; Figure S8: Statistical graph of cell migration distance obtained according to Figure S7; Figure S9: Serum biochemistry assay of mice injected with or without MIL-101/ACF@CCM; Figure S10: In vitro hemolysis with different concentrations of MIL-101/ACF@CCM; Figure S11: The quantitation of fluorescence from each organ ex vivo (n = 3); Figure S12: The tumor weights of excised tumors at the end of treatment after (n = 6).
