# Supplementary material for: Cell-Membrane-Coated Metal–Organic Framework Nanocarrier Combining Chemodynamic Therapy for the Inhibition of Hepatocellular Carcinoma Proliferation
Source: Pharmaceutics. 2024 May 5;16(5):619. doi: 10.3390/pharmaceutics16050619 (PMC11124917; doi:10.3390/pharmaceutics16050619)
Supplement: Supplementary file 1 [file pharmaceutics-16-00619-s001.zip › pharmaceutics-2946335-supplementary.pdf]

Supporting Information

Cell membrane-coated MOF nanocarrier combining CDT for inhibition of hepatocellular carcinoma proliferation and metastasis

Huaying Xie [1,†], Xuhua Xiao [2,†], Xiaoyuan Yi[1], Kunzhao Huang[1], Qingyu Zeng[1], Feifei He[2], Liyan Wang[2,*]

1 The First School of Clinical Medicine, Guilin Medical University, Guilin 541000, P.R. China.

2 Department of Gastroenterology, Affiliated Hospital of Guilin Medical University, Guilin 541000, P.R. China.

* Correspondence: 168wangliyan@163.com; Tel.: 15295953938.

† These authors contributed equally to this work.

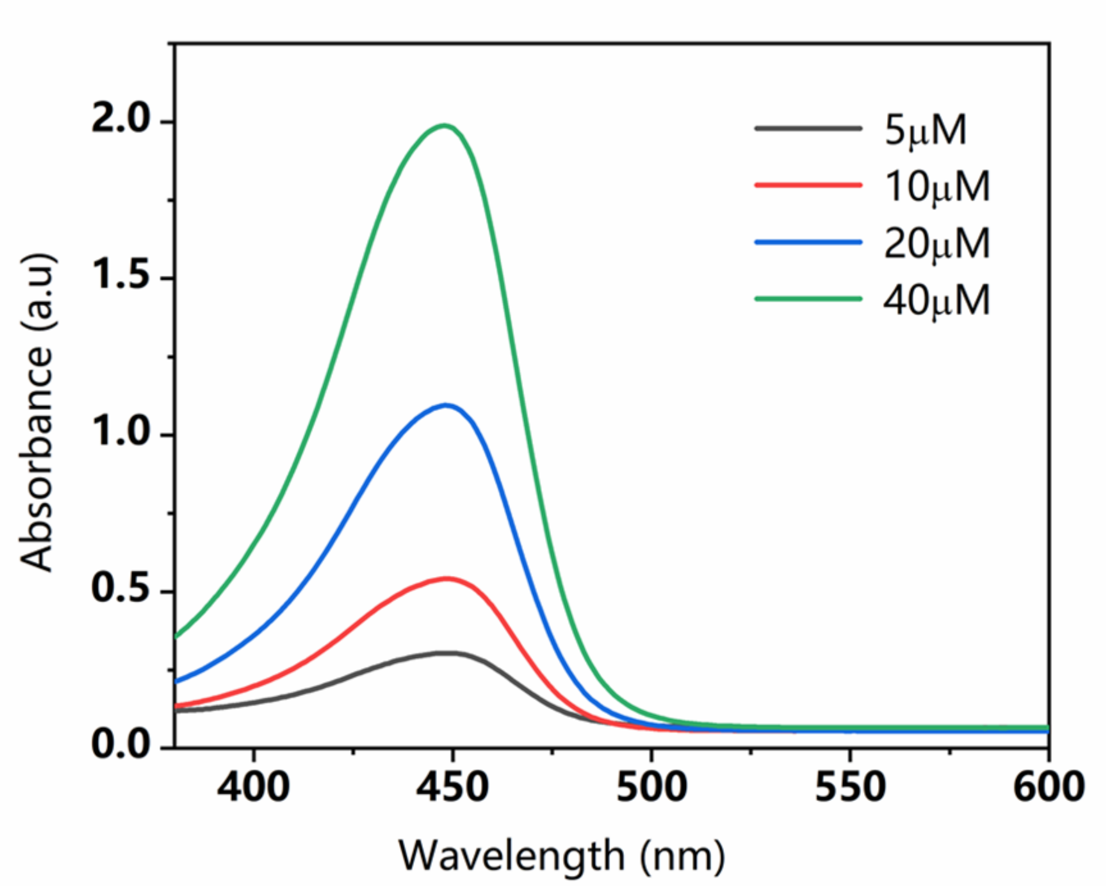

Figure S1. UV-vis absorption spectra of ACF in H_2O solution at different concentrations.

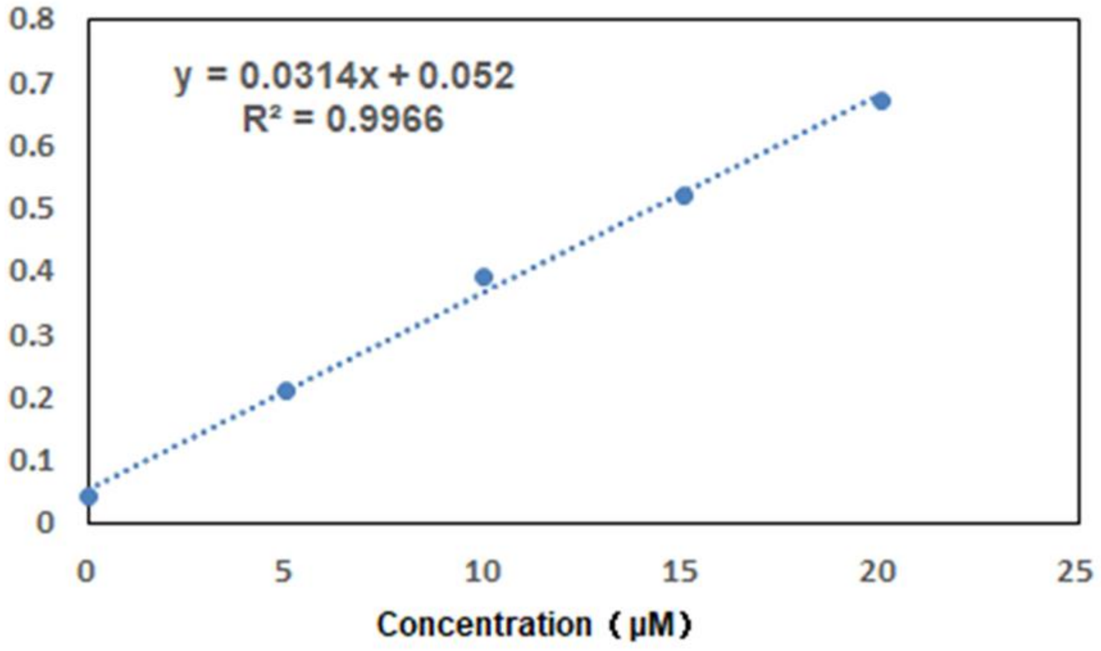

Figure S2. The linearly fitted standard curve of ACF characteristic absorption intensity at 460 nm vs concentration.

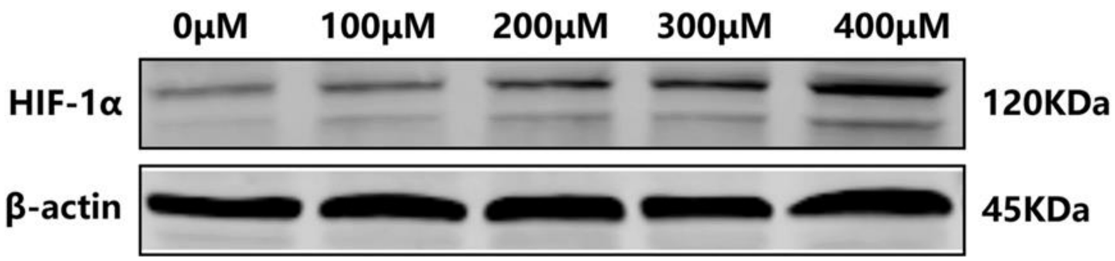

Figure S3. Western blot bands of hypoxia-related proteins (i.e., HIF-1α) in Hepa1-6 cells after incubating with $CoCl_2$ with varied concentrations.

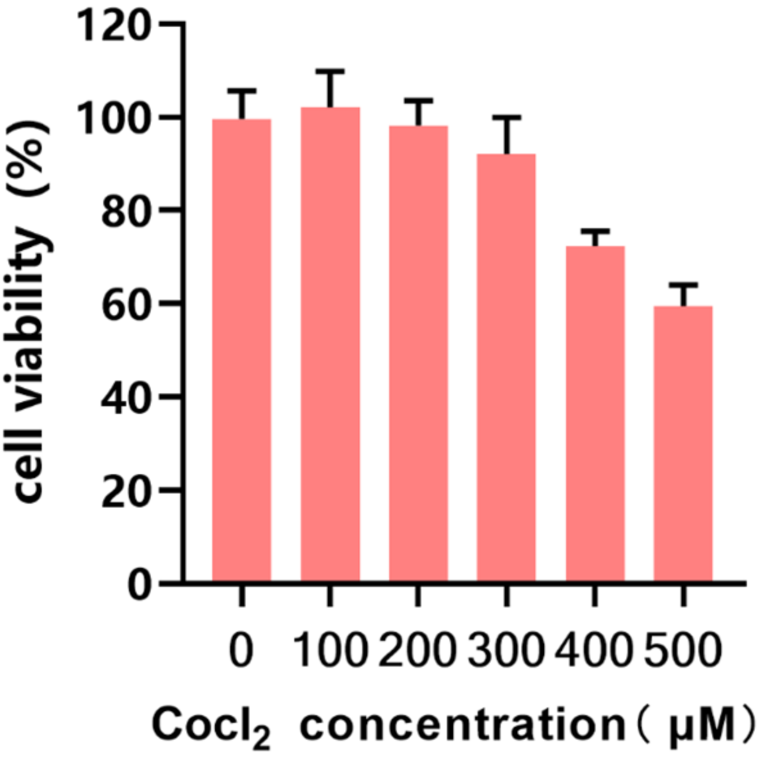

Figure S4. Cell viability of Hepa1-6 cells after incubating with $CoCl_2$ with varied concentrations.

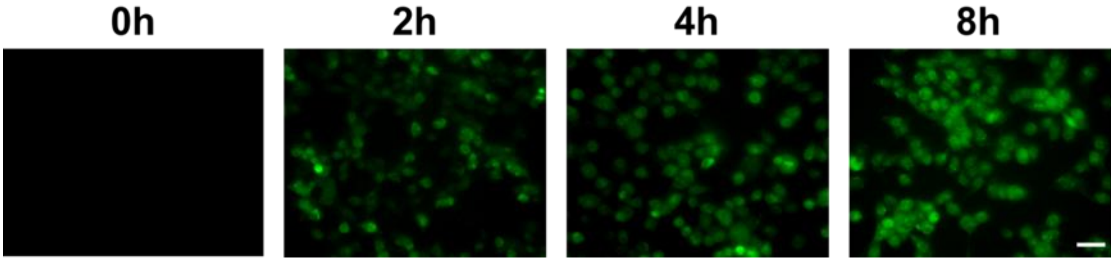

Figure S5. Cell internalization of MIL-101/ACF@CCM nanoagents by Hepa1-6 cells.

Scale bar: 50 μm.

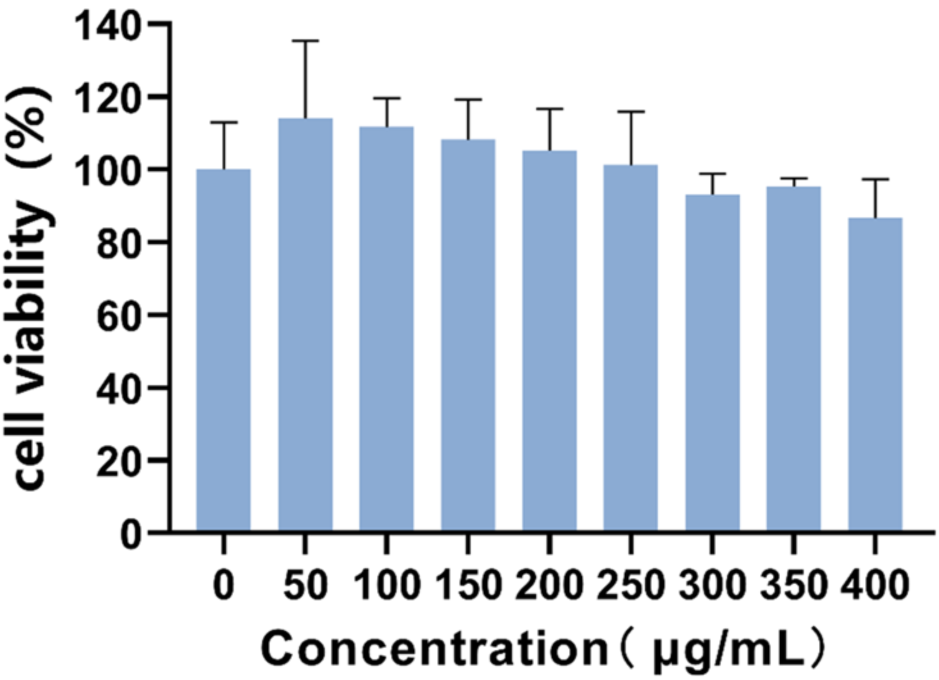

Figure S6. Cell viabilities of THLE-2 cells after incubation with varied concentrations of MIL-101/ACF@CCM nanoagents for 24 h.

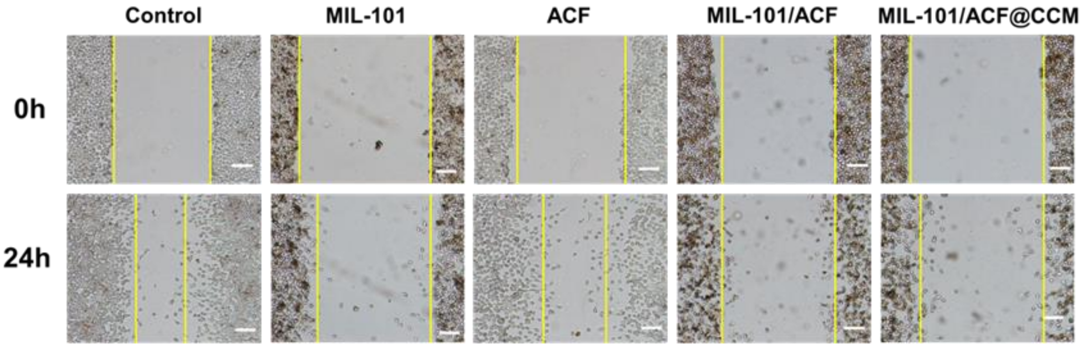

Figure S7. Images of wound healing of hypoxic Hepa1-6 cells after different treatments.

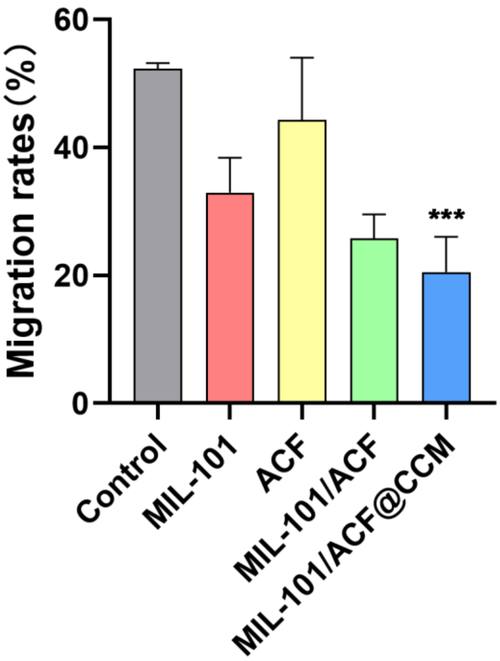

Figure S8. Statistical graph of cell migration distance obtained according to Figure S7. n=3. ***$p < 0.001$.

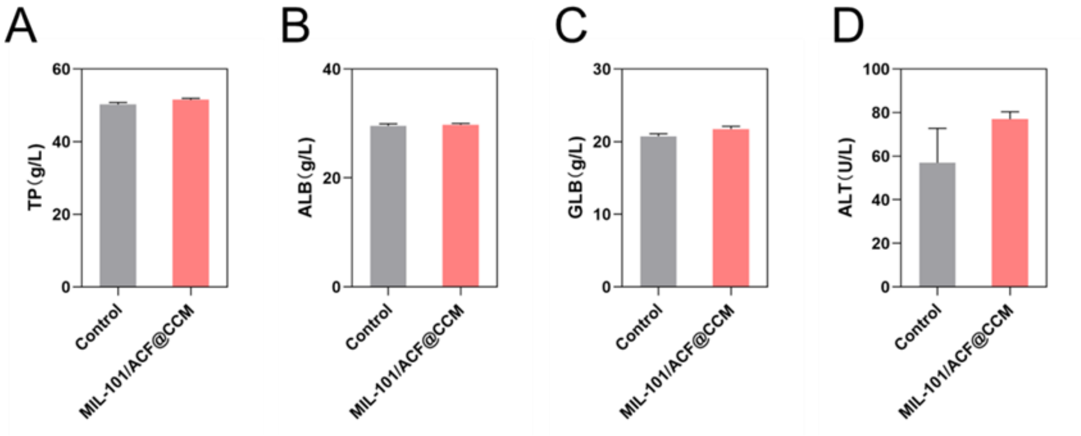

Figure S9. Serum biochemistry assay of mice injected with or without MIL-101/ACF@CCM.

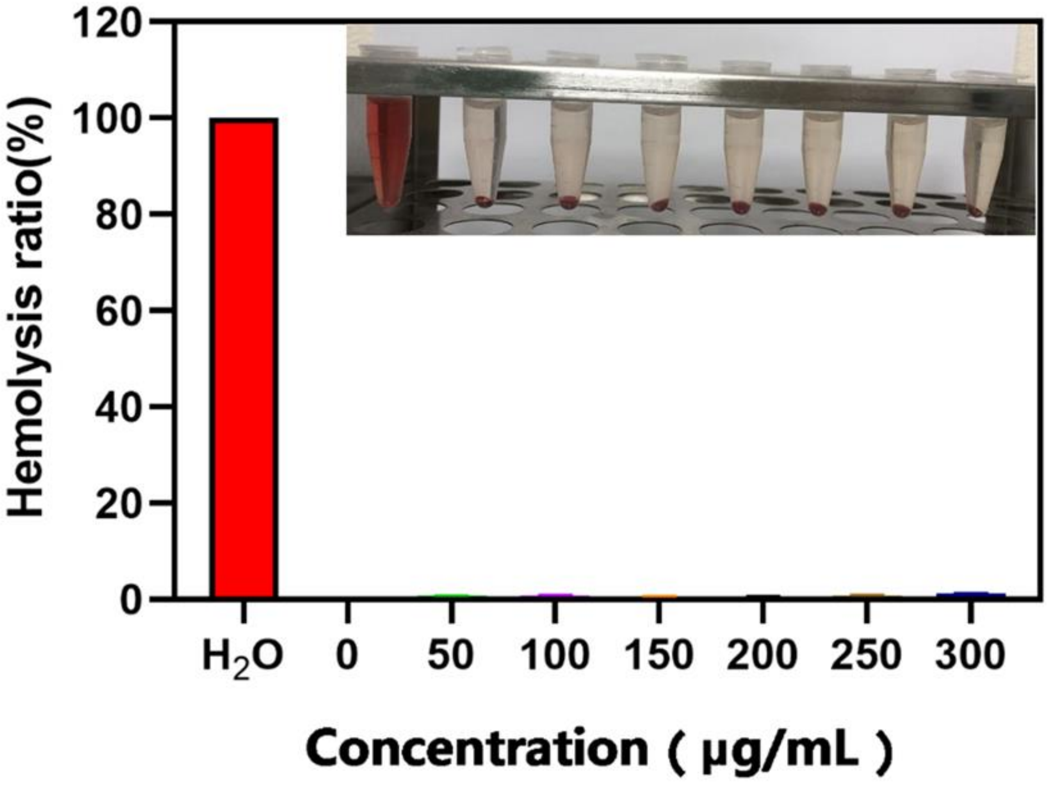

Figure S10. In vitro hemolysis with different concentrations of MIL-101/ACF@CCM.

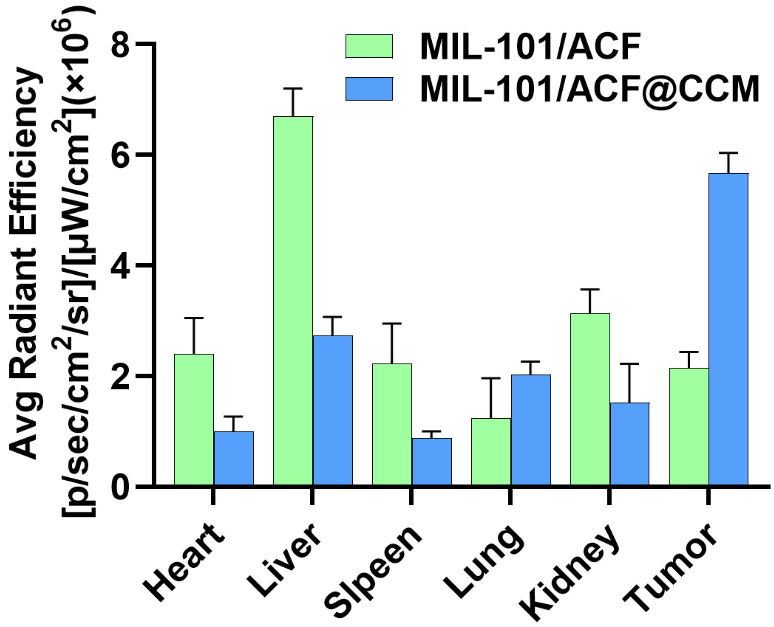

Figure S11. The quantitation of fluorescence from each organ ex vivo (n = 3).

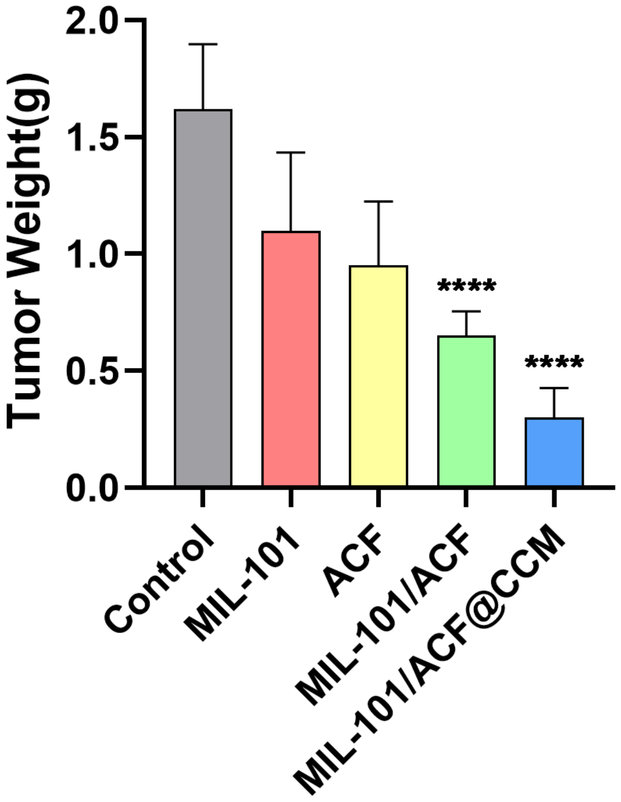

Figure S12. The tumor weights of excised tumors at the end of treatment after (n = 6). ****$p < 0.0001$.
